# Supplementary material for: Two types of motifs enhance human recall and generalization of long sequences
Source: Commun Psychol. 2025 Jan 7;3:3. doi: 10.1038/s44271-024-00180-8 (PMC11707037; doi:10.1038/s44271-024-00180-8)
Supplement: Supplementary file 2 — supplementary information [file 44271_2024_180_MOESM2_ESM.pdf]

Supplementary References

0.1 Experiment 1

0.1.1 Training

Regression Coefficient Other regressors that showed significant effects are serial position, trial ID, chunk boundary, and the number of repetitions. Serial position is the n-th item recalled in a trial, significantly affecting recall correctness ($\chi^2(1) = 697.92$, $p < 0.001$). The further the position of a sequence recall, the more likely that participants will be making a mistake ($\beta = -0.29$, $se = 0.03$, $z = -11.60$, $p < 0.001$). This result is consistent with the primacy effect widely observed in the serial recall literature[1] as mistake probability increases with the serial position. Apart from that, trial ID, i.e., the number of practice trials ($\chi^2(1) = 810.02$, $p < 0.001$), also increases the log-odds of recalling correctly ($\beta = 0.08$, $se = 0.02$, $z = 4.83$, $p < 0.001$), confirming a practice effect over training blocks.

We also observed that the sub-sequence boundary (at the first, fourth, fifth, eighth, ninth, and twelfth item of the sequence) affects recall correctness ($\chi^2(1) = 34.767, p < 0.001$). Items located at the beginning and the end of the displayed sub-sequence are more likely to be recalled correctly compared to the items within each sub-sequence ($\beta = 0.15$, $se = 0.02$, $z = 6.64$, $p < 0.001$). This observation resonates with the literature suggesting participants have more accurate memory and recall performance at the boundaries of serially ordered sub-sequences than between[2–4]. Additionally, the number of exact repetitions ($\chi^2(1) = 158.19, p < 0.01$) increases the log odds of correct recall press ($\beta = 0.07$, $se = 0.01$, $z = 5.10$, $p < 0.001$).

0.1.2 Transfer

Regression Coefficient Apart from transfer types, the recall keypress correctness decreases with the recall sequence position ($\chi^2(1) = 322.3$, $p < 0.001$). The further participants are into recall, the more likely they will make mistakes ($\beta = -0.08$, $\sigma = 0.008$, $z = -10.78$, $p < 0.001$). The decrease in recall accuracy is consistent with the primacy effect in memory literature: items that occur early in a sequence tend to be remembered and recalled more accurately[1]. We also observed a practice effect: trial ID affects the log odd ratio of pressing the right key ($\chi^2(1) = 86.07, p < 0.001$) ($\beta = 0.09$, $se = 0.01$, $z = 5.94$, $p < 0.001$). Apart from that, chunk boundary effect was also observed: the subchunk boundaries generally exhibit a higher recall accuracy than the interchunk items ($\chi^2(1) = 25.17, p < 0.001$) ($\beta = 0.17$, $\sigma = 0.03$, $z = 5.17$, $p < 0.001$), resonating with existing findings that chunk boundaries are remembered more accurately than within-chunk items[3].

0.2 Experiment 2

0.2.1 Training

Regression Coefficient Other regressors that showed significant effects are serial position ($\beta = -0.68$, $se = 0.04$, $z = -16.37$, $p < 0.001$, 95% CI = -0.77 to -0.60), confirming the recency effect; Trial ID ($\beta = 0.64$, $se = 0.13$, $z = 5.04$, $p < 0.001$), confirming the practice effect; the number of repetitions ($\beta = 0.05$, $se = 0.01$, $z = 4.41$, $p < 0.001$); and chunk boundary ($\beta = 0.32$ $se = 0.02$, $z = 13.29$, $p < 0.001$).

0.2.2 Transfer

Regression Coefficient Similar to the training block, we observed a recency effect ($\beta = -0.64$, $se = 0.02$, $z = -31.09$, $p < 2e-16$, 95% CI = -0.69 to -0.61), practice effect ($\beta = 0.32$, $se = 0.02$, $z = 13.30$, $p < 0.001$, 95% CI = 0.28 to 0.37), repetition effect ($\beta = 0.05$, $se = 0.03$, $z = 1.85$, $p = 0.06$, 95% CI = 0.00 to 0.12), and chunk boundary effect($\beta = 0.31$, $se = 0.04$, $z = 7.91$, $p < 0.001$, 95% CI = 0.24 to 0.39), confirming a viable expectation over experimental manipulation.

0.3 Reaction Time Analysis

As shown in Supplementary Figure S1 average reaction time for the three training groups decreases with practice, and reaction time converges at the end of the training block for all three groups. Supplementary Figure S1 b shows the reaction time to press the recall sequence within each recall trial. Shown in Supplementary Figure S1 c is the average reaction time across the three training groups. The average reaction time to recall the sequence does not differ significantly amongst the three groups, as indicated via fitting a linear mixed effect regression model onto participants' recall time, assuming a random intercept over individual participants and a random slope over serial positions ($\chi^2(2) = 4.32$, $p = .11$).

Other regressors that showed significant effects are serial position, trial ID, chunk boundary, and repetitions, as shown in Supplementary Figure S2. Serial position, the n-th item recalled in a trial, affects reaction time. The further the position of a sequence recall, the shorter the reaction time ($\beta = -126.76$, $se = 20.51$, $t = 103.30$, $p < 0.001$). Trial ID, i.e., the number of practice trials, also reduces reaction time ($\beta = -117.413$, $se = 5.86$, $t = -20$, $p < 0.001$), confirming a practice effect over the training phase. Immediate repetitions of the previous sequence also drives reaction time faster ($\beta = -61.86$, $se = 9.28$, $t = -6.67$, $p < 0.001$). Reaction time of the first item in each subsequence position is much higher than other serial positions in the sequence ($\beta = 573.54$, $se = 7.19$, $t = 79.73$, $p < 0.001$), reflecting the structure of the task.
Shown in Supplementary Figure S1 d is the average reaction time during the transfer phase: for the groups trained on motifs, transfer type affects their transfer performance ($\chi^2 = 174.05$, $p < 0.001$). When the motif groups transfer to the test blocks,

their reaction time to recall the sequence and execute the sequence presses is higher for transferring to the same motif compared to transferring to an independent block ($\hat{\beta} = -109.34$, $se = 9.49$, $t(30417) = -11.514$, $p =< 0.0001$). When the motif group transfers to a different motif, the reaction time speed up is not significantly higher than the transfer to an independent block ($\hat{\beta} = -2.22$, $se = 9.49$, $t(30417) = -0.23$, $p = 0.81$). Having trained on sequences with motifs, participants recall sequences faster when transferring to a sequence with the same motif but not necessarily to a different motif.
For experiment 2, we also fitted a linear mixed effect regression model onto participants' recall time, assuming a random intercept over individual participants and a random slope over trial ID. As shown in Supplementary Figure S3, regressors that showed significant effects during the training block are serial position, trial ID, and chunk boundary. Serial position, the n-th item recalled in a trial, affects reaction time. The further the position of a sequence recall, the shorter the reaction time ($\beta = -97.68$, $se = 4.09$, $t = -23.84$, $p < 0.001$). Trial ID, i.e., the number of practice trials, also reduces reaction time ($\beta = -134.271$, $se = 19.62$, $t = -6.84$, $p < 0.001$), confirming a practice effect over the training phase. Number of repetitions drives reaction time faster ($\beta = -7.15$, $se = 2.23$, $t = -3.20$, $p = 0.002$). Reaction time of the first item in each subsequence position is much higher than other serial positions in the sequence ($\beta = 653.003$, $se = 9.46$, $t = 69.02$, $p < 0.001$), reflecting the structure of the task.
During the transfer phase, a linear mixed effect regression on recall time, assuming a random intercept over individual participants and a random slope over trial ID and serial position shows serial position ($\beta = -140.66$, $se = 19.02$, $t = -7.39$, $p < 0.001$), and chunk boundary as affecting the reaction time ($\beta = 773.94$, $se = 18.95$, $t = 40.85$, $p < 0.001$).

0.4 Model Specification

Algorithm 1 Motif Learning

Require: seq: learning sequences
Require: cg: dictionary of learned chunks
Require: $threshold\_chunk$: boolean flag for learning chunks
Require: $abstraction$: boolean flag for learning variables

```
chunk_record ← {}                                              ▷ Initialize chunk record
t ← 0
while not seq_over do
    current_chunks, cg, seq, chunk_record ← identify_latest_chunks(cg, seq)
    cg ← learning_and_update(current_chunk, chunk_record, cg, threshold_chunk = True)
    if abstraction then
        cg ← abstraction_update(current_chunks, cg)
    end if
    cg.forget()                                    ▷ multiple all frequency record by θ
end while
return cg, chunk_record
```

The model initiates with a dictionary cg, which holds chunks (sub-sequences) and the transition between chunks. When an instruction sequence is presented to the model, it consecutively parses the sequence via the chunks in the dictionary that contain the biggest size. At each parsing step, the model updates the frequencies of each parsed item and transition frequencies between the previously parsed item and the current one. After parsing a chunk, the boolean flag $thresholdchunk$ and $abstraction$ control the model to create new chunks or to learn new variables.
$thresholdchunk$ is a boolean flag that indicates whether the algorithm will learn and combine new chunks based on the input sequence (True) or just parse the sequence with existing items in the dictionary (False). In case it is true, then the algorithm checks if the currently identified chunk and the previously identified chunk have been conjunctively activated more than a minimum threshold in the transition matrix ($N = 3$). On top of that, a hypothesis test (χ^2) is conducted to assess whether the consecutively parsed chunks are correlated with significance level $p < 0.05$. If so, then a new chunk is created by combining the previous with the current and incorporating it into the chunking graph cg. This procedure also includes cases where the current chunk contains variables within.
$abstraction$ is another boolean flag that controls the learning of variables. When this flag is on, the model constructs new variables from chunks that share common ancestors and common descendants, indicating these chunks share similar occurrence contexts. A new variable is created if it connects a set of chunks with a combined frequency above a threshold ($freq_T = 6$). At the end of each sequence parse, the algorithm performs a "forgetting" step, which multiplies all chunk occurrences and transition frequencies by $\theta = 0.996$.

Abstraction Learning When simulating learning projectional motifs, we turn on the $thresholdchunk$ and learn sequences

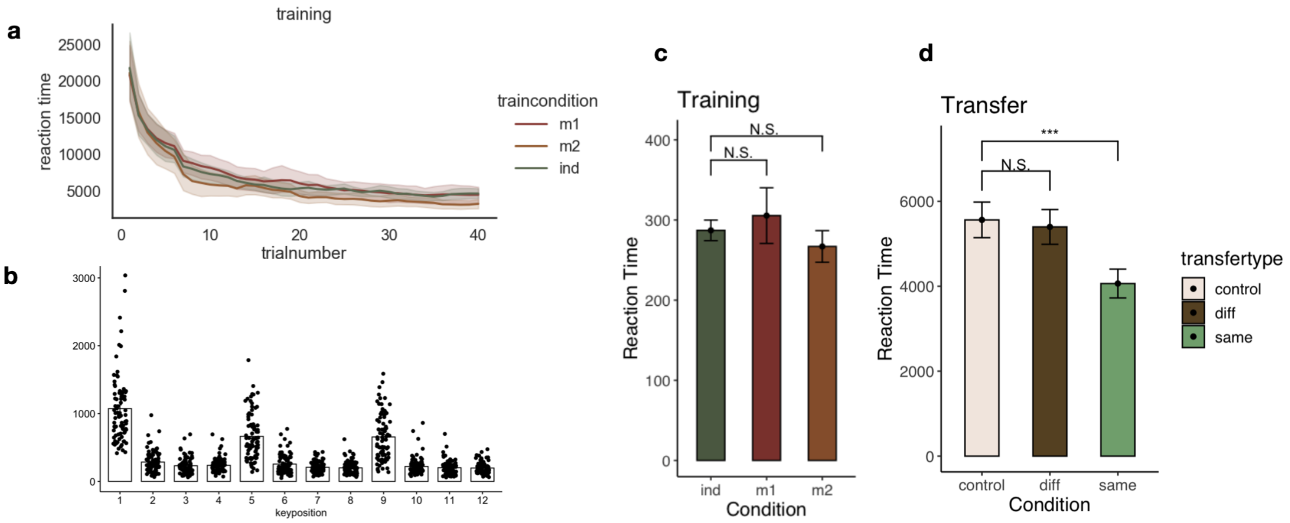

Figure S1. Reaction time analysis. a. Average reaction time across training trials. b. Average reaction time across recall sequence position. c. Average reaction time during the training block. d. Average reaction time during the transfer block across three transfer types. Same: Motif 1 – Motif 1 and Motif 2 – Motif 2; different: Motif 1 – Motif 2, and Motif 2 – Motif 1; control: Independent – Motif 1, and Independent – Motif 2.

on the projectional motif space. When simulating learning variable motifs, we set both the *learn* and *abstraction* flag to be true.
Chunking When simulating the chunking model, we turn on the *thresholdchunk* flag and turn off the *abstraction* flag.
Associative Learning When simulating the associative learning model, we turn off both the *thresholdchunk* flag and the *abstraction* flag. Thereby, no new chunks are created, and the model will learn the transition and occurrence frequencies of the atomic sequential elements.
Recall The recall function simulates the process of sequential recall from a chunk graph, starting with a primed item and proceeding through associative transitions. Given a priming first item of the sequence, the model samples a chunk consistent with the primed first item. Subsequent chunks are sampled based on transition probabilities from the previously recalled chunk (prev). The process repeats until the length of the recalled sequence reaches the desired sequence length $seql = 12$.

Supplementary References

1. Oberauer, K. Understanding serial position curves in short-term recognition and recall. *J. Mem. Lang.* **49**, 469–483, DOI: https://doi.org/10.1016/S0749-596X(03)00080-9 (2003).

2. Lewandowsky, S. & Jr, B. Memory for serial order. *Psychol. Rev.* **96**, 25–57, DOI: 10.1037/0033-295X.96.1.25 (1989).

3. Farrell, S. Temporal clustering and sequencing in short-term memory and episodic memory. *Psychol. Rev.* **119**, 223–271, DOI: 10.1037/a0027371 (2012).

4. Cowan, N., Saults, J., Elliott, E. M. & Moreno, M. V. Deconfounding Serial Recall. *J. Mem. Lang.* **46**, 153–177, DOI: 10.1006/jmla.2001.2805 (2002).

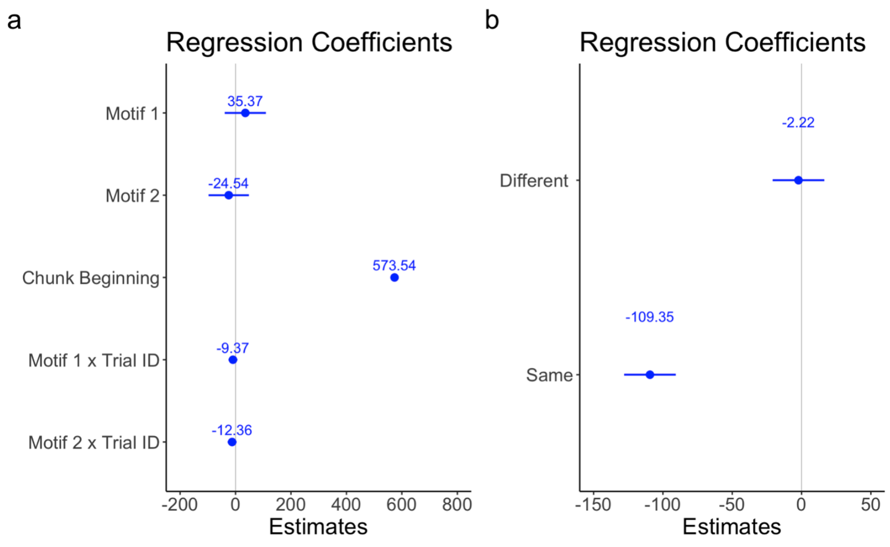

Figure S2. Reaction time analysis. a. regression coefficient of experiment 1 during training. b. transfer

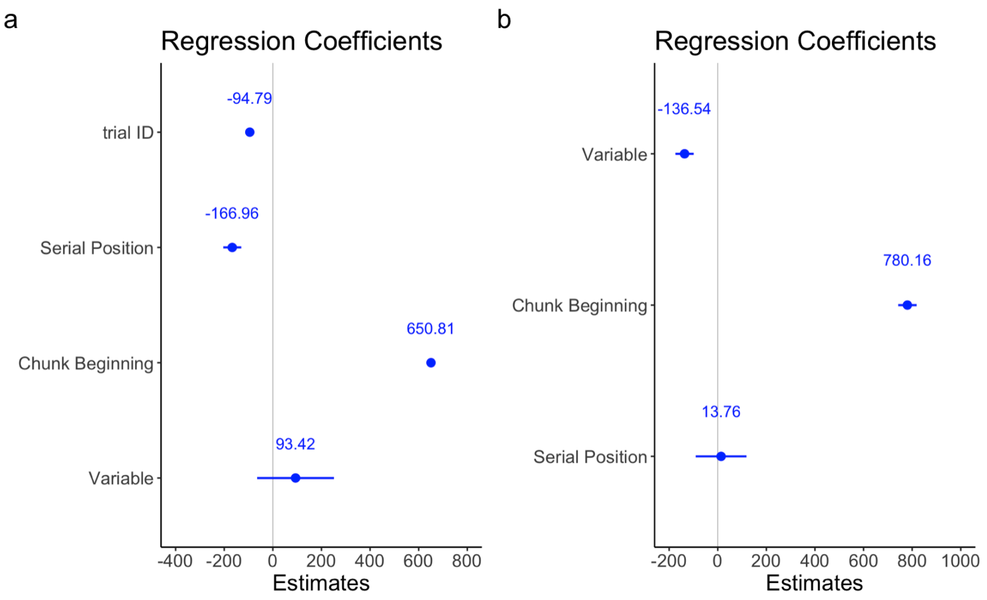

Figure S3. Reaction time analysis. a. regression coefficient of experiment 2 during training. b. transfer
