# Supplementary material for: Two types of motifs enhance human recall and generalization of long sequences
Source: Commun Psychol. 2025 Jan 7;3:3. doi: 10.1038/s44271-024-00180-8 (PMC11707037; doi:10.1038/s44271-024-00180-8)
Supplement: Supplementary file 3 — reporting summary [file 44271_2024_180_MOESM3_ESM.pdf]

Corresponding author(s): *Double-anonymous peer review submissions: write DAPR and your manuscript number here instead of author names.*

Last updated by author(s): *YYYY-MM-DD*

Reporting Summary

Nature Portfolio wishes to improve the reproducibility of the work that we publish. This form provides structure for consistency and transparency in reporting. For further information on Nature Portfolio policies, see our Editorial Policies and the Editorial Policy Checklist.

Statistics

For all statistical analyses, confirm that the following items are present in the figure legend, table legend, main text, or Methods section.

n/a	Confirmed	
☐	☒	The exact sample size (*n*) for each experimental group/condition, given as a discrete number and unit of measurement
☐	☒	A statement on whether measurements were taken from distinct samples or whether the same sample was measured repeatedly
☐	☒	The statistical test(s) used AND whether they are one- or two-sided<br>*Only common tests should be described solely by name; describe more complex techniques in the Methods section.*
☐	☒	A description of all covariates tested
☐	☒	A description of any assumptions or corrections, such as tests of normality and adjustment for multiple comparisons
☐	☒	A full description of the statistical parameters including central tendency (e.g. means) or other basic estimates (e.g. regression coefficient) AND variation (e.g. standard deviation) or associated estimates of uncertainty (e.g. confidence intervals)
☐	☒	For null hypothesis testing, the test statistic (e.g. *F*, *t*, *r*) with confidence intervals, effect sizes, degrees of freedom and *P* value noted<br>*Give P values as exact values whenever suitable.*
☒	☐	For Bayesian analysis, information on the choice of priors and Markov chain Monte Carlo settings
☐	☒	For hierarchical and complex designs, identification of the appropriate level for tests and full reporting of outcomes
☐	☒	Estimates of effect sizes (e.g. Cohen's *d*, Pearson's *r*), indicating how they were calculated

Our web collection on statistics for biologists contains articles on many of the points above.

Software and code

Policy information about availability of computer code

Data collection	Data is collected via participants interacting with a programmed web experiment provided on the Prolific platform. The experimental code is available and uses all open-source packages in the GitHub repository: https://github.com/swu32/motif_learning
Data analysis	The data analysis is conducted using the software package R, and the simulation is conducted in Python. Both are freely available.

For manuscripts utilizing custom algorithms or software that are central to the research but not yet described in published literature, software must be made available to editors and reviewers. We strongly encourage code deposition in a community repository (e.g. GitHub). See the Nature Portfolio guidelines for submitting code & software for further information.

Data

Policy information about availability of data

All manuscripts must include a data availability statement. This statement should provide the following information, where applicable:

- Accession codes, unique identifiers, or web links for publicly available datasets
- A description of any restrictions on data availability
- For clinical datasets or third party data, please ensure that the statement adheres to our policy

The data collected in this experiment is publicly available under the link: https://github.com/swu32/motif_learning

Research involving human participants, their data, or biological material

Policy information about studies with human participants or human data. See also policy information about sex, gender (identity/presentation), and sexual orientation and race, ethnicity and racism.

Reporting on sex and gender	We recruited 135 participants for Experiment 1 from Prolific, an online crowd-sourcing experimental platform. Out of all participants, thirty-seven were female, ninety-eight were male. We recruited 120 participants for Experiment 2 from Prolific, out of which thirty-four were female, eighty-six were male. The findings are not specific to any particular sex or gender.
Reporting on race, ethnicity, or other socially relevant groupings	The study does not group participants based on race, ethnicity, or other social categorizations.
Population characteristics	In experiment 1, participants' ages ranged from 18 to 67, with an average of 32 and a median of 28. In experiment 2, participants' ages ranged from 19 to 63, with an average of 31.2 and a median of 28.
Recruitment	Participants are recruited from all eligible participant pool available from the Prolific platform.
Ethics oversight	The experiments were performed following the relevant guidelines and regulations approved by the ethics committee of the University of Tuebingen (Ethik-Kommission an der Medizinischen Fakultät der Eberhard-Karls-Universität und am Universitätsklinikum Tübingen), under the study title: Experimente zum Sequenz- und Belohnungslernen, with application number 701/2020BO.

Note that full information on the approval of the study protocol must also be provided in the manuscript.

Field-specific reporting

Please select the one below that is the best fit for your research. If you are not sure, read the appropriate sections before making your selection.

☐ Life sciences ☒ Behavioural & social sciences ☐ Ecological, evolutionary & environmental sciences

For a reference copy of the document with all sections, see nature.com/documents/nr-reporting-summary-flat.pdf

Behavioural & social sciences study design

All studies must disclose on these points even when the disclosure is negative.

Study description	The study includes quantitative data that describes recall accuracy and reaction time from participants.
Research sample	The research sample comes from the available pool of participants on Prolific, an online crowd-sourcing experimental platform. In Experiment 1, 135 participants were recruited, thirty-seven were female. Participants' ages ranged from 18 to 67, with an average of 32 and a median of 28. In Experiment 2, 120 participants were recruited, out of which thirty-four were female. Participants' ages ranged from 19 to 63, with an average of 31.2 and a median of 28. The research sample is a representative sample from the available pool of participants on Prolific.
Sampling strategy	We did not perform sample size calculation.
Data collection	Data was collected through participants' interaction with our programmed experimental webpage, which records participants' keypresses and their reaction time. Researchers are blind to the specific experimental condition of individual participants.
Timing	We collected data from 45 participants in experiment 1 between in June 2022, another 90 participants were collected in May 2023. Experiment 2's data was collected from December 2022 until June 2023 in multiple batches.
Data exclusions	For both experiment, we applied exclusion criteria based on reaction time, recall accuracy, and learning curve of participants. In experiment 1, after filtering, 37 participants are left in group m1, 41 in m2, and 28 in group independent.<br>In experiment 2, after filtering, 45 participants out of 120 remained in group m1, and 52 remained in group control.
Non-participation	In Experiment 1, 28 participants withdrew from the study and 3 timed out. 19 participants withdrew from Experiment 2 and 4 timed out. Multiple factors can contribute to an earlier study departure, which includes unintended participation, distraction, or technical problems encountered on individual devices.
Randomization	Participants who previously have participated in the study are excluded from a second-time participation.

Reporting for specific materials, systems and methods

We require information from authors about some types of materials, experimental systems and methods used in many studies. Here, indicate whether each material, system or method listed is relevant to your study. If you are not sure if a list item applies to your research, read the appropriate section before selecting a response.

Materials & experimental systems

n/a	Involved in the study
☒	☐ Antibodies
☒	☐ Eukaryotic cell lines
☒	☐ Palaeontology and archaeology
☒	☐ Animals and other organisms
☒	☐ Clinical data
☒	☐ Dual use research of concern
☒	☐ Plants

Methods

n/a	Involved in the study
☒	☐ ChIP-seq
☒	☐ Flow cytometry
☒	☐ MRI-based neuroimaging

Plants

Seed stocks	*Report on the source of all seed stocks or other plant material used. If applicable, state the seed stock centre and catalogue number. If plant specimens were collected from the field, describe the collection location, date and sampling procedures.*
Novel plant genotypes	*Describe the methods by which all novel plant genotypes were produced. This includes those generated by transgenic approaches, gene editing, chemical/radiation-based mutagenesis and hybridization. For transgenic lines, describe the transformation method, the number of independent lines analyzed and the generation upon which experiments were performed. For gene-edited lines, describe the editor used, the endogenous sequence targeted for editing, the targeting guide RNA sequence (if applicable) and how the editor was applied.*
Authentication	*Describe any authentication procedures for each seed stock used or novel genotype generated. Describe any experiments used to assess the effect of a mutation and, where applicable, how potential secondary effects (e.g. second site T-DNA insertions, mosiacism, off-target gene editing) were examined.*
